# The New Double‐row Bankart Repair Recovered Shoulder Stability without Excessive Motion Limitation: A Case–Control Study with Single‐row Bankart Repair

**DOI:** 10.1111/os.14032

**Published:** 2024-03-15

**Authors:** Xu Cheng, Hangle Wang, Yanfang Jiang, Zhenxing Shao, Guoqing Cui

**Affiliations:** ^1^ Department of Sports Medicine Peking University Third Hospital, Institute of Sports Medicine of Peking University Beijing China; ^2^ Beijing Key Laboratory of Sports Injuries Beijing China; ^3^ Engineering Research Center of Sports Trauma Treatment Technology and Devices, Ministry of Education Beijing China

**Keywords:** Anterior shoulder instability, Bankart repair, Double‐row, Single‐row, Surgical technique

## Abstract

**Objectives:**

Bankart lesion is one of the most common lesions of the glenohumeral joint. Several double‐row suture methods were reported for Bankart repair, which could provide more stability, yet more motion limitation and complications. Therefore, we introduced a new double‐row Bankart repair technique, key point double‐row suture which used one anchor in the medial line. The purpose of this article is to investigate the clinical outcomes of this new method and to compare it with single‐row suture.

**Methods:**

Seventy‐eight patients receiving key point double‐row suture or single‐row suture from October 2010 to June 2014 were collected retrospectively. The basic information including gender, age, dominant arm, and number of episodes of instability was collected. Before surgery, the glenoid bone loss was measured from the CT scan. The visual analogue scale, American shoulder and elbow surgeons, the University of California at Los Angeles shoulder scale, and subjective shoulder value were valued before surgery and at the last follow‐up.

**Results:**

Forty‐four patients (24 patients receiving single‐row suture and 20 patients receiving key point double‐row suture) were followed up successfully. The follow‐up period was 9.2 ± 1.1 years (range, 7.8–11.4 years). At the last follow‐up, no significant differences were detected for any of the clinical scores. The recurrence rate was 12.5% for the single‐row group and 10% for the double‐row group, respectively (*p* = 0.795) 14 patients (31.8%) in the single‐row group and nine patients (26.5%) in the double‐row group were tested for active range of motion. A statistically significant difference was found only for the internal rotation at 90° abduction (48.9° for single‐row and 76.7° for key point double‐row, *p* = 0.033).

**Conclusion:**

The key point double‐row sutures for Bankart lesions could achieve similar long‐term outcomes compared with single‐row suture, and one medial anchor did not result in a limited range of motion. The low recurrence rate and previous biomechanical results also indicate the key point double‐row suture is a reliable method.

## Graphical abstract

The key point double‐row suture technique, as compared to the single‐row suture technique, provides an additional medial anchor without any significant reduction in the range of motion. The biomechanical results and low recurrence rate of the key point double‐row suture technique further support its reliability and effectiveness in Bankart repair.

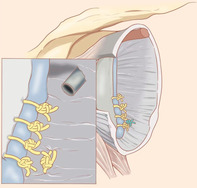

## Introduction

The capsulolabral complex, composed of the glenohumeral ligament and capsule, is an important soft structure, providing stabilization when the humerus shifts anteriorly. On the contrary, when the shoulder dislocates anteriorly where the cuff muscles are also weak, the capsulolabral complex is easily torn off, that is, a Bankart lesion has occurred. Since this lesion was described by Bankart^1^ in 1938, it has been one of the common pathologies in shoulder lesions, which causes 7.9%–50.0% of the traumatic glenohumeral instability.^2^ So, trying to fix it is critical to restoring the stability of the shoulder joint.

The repair of a Bankart lesion is reattaching the capsulolabral complex to the rim of the glenoid, which is usually performed arthroscopically. Porcellini *et al*.^3^ first published the results of the arthroscopic Bankart repair in 25 patients with at least a 2‐year follow‐up, and most patients restored function and stability. The Bankart repair could be divided into single‐row and double‐row according to the configuration of suture anchors. Lafosse *et al*.^4^ invented the double‐row repair method, which was called Cassiopeia double‐row technique in their article. He also reported the short‐term results in 12 patients, and there were no complications or recurrence of instability. After that, many biomechanical studies confirmed the advantages of double‐row repair. Ahmad *et al*.^5^ found that double‐row repair got a larger footprint area providing the basis of healing. Kim *et al*.^6^ reported stress could achieve broader area and even distribution in double‐row repair. Spiegl *et al*.^2^ concluded that the shoulder with a double‐row repair could sustain a greater load. Although McDonald *et al*.^7^ reported the double‐row repair resulted in more external rotation loss, the kinematics test still supported double‐row repair.

In short, the clinical results of single‐row Bankart repair were still unsatisfied, while the double‐row suture was short of clinical data. To combine the advantages of both methods, we created a brand‐new suture method based on traditional double‐row repair—key point double‐row repair, which just uses one anchor in the medial line. Our study aims to: (i) introduce the new suturing method; (ii) evaluate the efficacy of key point double‐row repair; and (iii) compare it with single‐row repair. Our hypothesis is key point double‐row repair could achieve clinical results no inferior to single‐row repair.

## Methods

### Study Design

This study received approval of the ethics committee before initiation (No. M2022685). The main steps included selecting patients, collecting data, and comparing differences. The patients' information was retrospectively obtained from recorded medical history and the personal and private information was concealed.

### Patient Selection

From October 2010 to June 2014, 78 patients with Bankart lesions received procedures in our hospital, 44 using single‐row repair and 34 using key point double‐row repair. The inclusion criteria were: (i) having a history of recurrent anterior shoulder instability (more than 1 instability episode); (ii) an arthroscopic exam confirmed there were Bankart lesions; and (iii) cooperating with the surgeries and rehabilitations. The exclusion criteria were: (i) having a history of epilepsy; (ii) posterior or multi‐directional instability; (iii) glenoid bone loss ≥25%; and (iv) arthroscopy found large Hill‐Sachs lesions (>25% of articular surface^8^). All procedures were performed by one experienced surgeon. The basic information was collected and compared between the two groups.

### Surgical Technique

The patients were placed in an oblique lateral decubitus position and anesthetized regularly. After checking the anterior shoulder instability test, Sulcus test, and laxity of every direction, the places of the acromion, coracoid, and acromioclavicular joint were marked. A standard posterior portal was used for a diagnostic arthroscopy, looking for synovium, Bankart lesions, glenohumeral joint, possible glenoid cartilage and bone loss, biceps tendon, subscapularis, posterior capsule, and possible Hill–Sachs lesions. The anterior superior portal and anterior inferior portal were established under arthroscopic observation. The arthroscopy was moved to the anterior superior portal for observation. Then using a periosteal stripper, the anteroinferior glenohumeral ligament and labrum complex were released across the glenoid rim and neck to 6 o'clock. Through the anterior inferior portal, three to four suture anchors were inserted at the edge of the glenoid, and sutures were threaded through the capsulolabral complex with a simple suture style by a lasso to knot.

**FIGURE 1:**
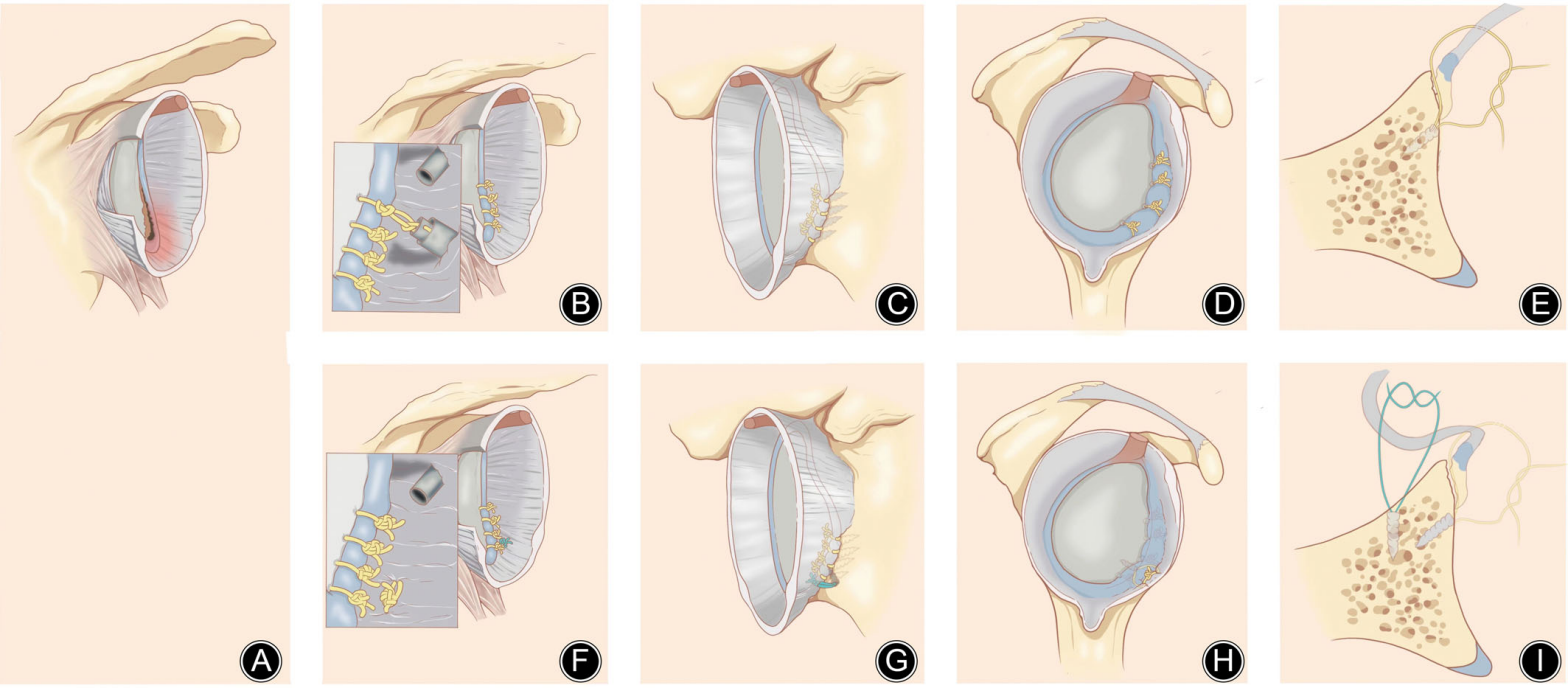
Comparison of single‐row and key point double‐row Bankart repair. (A) Bankart lesion; comparison of single‐row (B–E) and key point double‐row (F–I) Bankart repair in anterior aspect (B, F), posterior aspect (C, G), lateral aspect (D, H), and axial aspect (E, I).

The key point double‐row technique is similar to the single‐row. The difference was inserting an additional anchor in the 5'clock medial to glenoid surface 10 mm through the 5 o'clock portal. And sutured glenohumeral ligament and labrum complex with a mattress suture style (Figure 1).

### Post‐operative Protocol

Patients wore a shoulder sling with an abduction pillow for 6 weeks. One‐week post‐operation active‐assisted range of motion was progressed in forward flexion and external rotation. Combined abduction and external rotation were avoided until 12 weeks. Patients were allowed to return to sports at 6 months.

### Clinical Assignment

The visual analogue scale (VAS) for pain and instability, the rating scale of the American shoulder and elbow surgeons (ASES), the University of California at Los Angeles (UCLA) shoulder scale, and subjective shoulder value (SSV) were used to evaluate clinical outcomes. All scores were taken before surgery and at the last follow‐up. The glenoid bone loss before surgery was measured by CT scan and the value was obtained from the following equation: glenoid bone loss = (maximum defect width/diameter of the best‐fit circle) × 100%.

### Statistical Analysis

For dichotomous data, the chi‐square test was used to compare. For other data, the group *t*‐test was used for the comparison of normal distribution data, and the Mann–Whitney U test was used for non‐normal distribution data. All analyses were made with the Statistical Package for Social Science version 26. A significant difference was defined as *p* < 0.05.

## Results

### Baseline Information

Finally, 24 patients (54.5%) receiving single‐row suture and 20 patients (58.8%) receiving key point double‐row suture were followed up successfully. To eliminate the selective bias, the comparison between responders and non‐responders was summarized in Table 1, which did not show any difference except the gender. There were 12 females in the single‐row group and five females in the double‐row group. The follow‐up time for the single‐row group was 9.2 ± 1.1 years (range, 7.8–11.2 years), and for the double‐row group was 9.3 ± 1.2 years (range, 7.9–11.4 years). For the single‐row group, the mean age at first dislocation was 24.8 years old, and the mean age at operation was 31.1. For the double‐row group, these two ages were 20.3 and 26.7. The mean glenoid bone loss measured by CT scan before surgery was 10.7% for the single‐row group and 10.4% for the double‐row group. The preoperative clinical scores between the two groups did not show any difference. The demographics were shown in Table 2.

**TABLE 1:** Baseline information of responders and non‐responders (loss to follow‐up)

Baseline information	Responders (*n* = 44)	Non‐responders (*n* = 34)	*p* value
Gender (M/F, n)	27/17	31/3	0.003**
Age at first dislocation (years)	22.7 ± 8.1	23.0 ± 12.6	0.360
Age at operation (years)	28.7 ± 9.4	27.2 ± 12.1	0.117
Dominant arm (n)	31	19	0.183
Glenoid bone loss (%)	10.2 ± 7.8	7.4 ± 6.9	0.107
Number of episodes of instability	11.9 ± 16.8	10.1 ± 9.5	0.659
Forward flexion	174.5 ± 16.2	169.1 ± 20.7	0.142
External rotation at the side	44.8 ± 13.3	46.1 ± 10.0	0.534
Internal rotation at the side	68.6 ± 4.2	69.3 ± 7.8	0.976
External rotation at 90° abduction	88.1 ± 5.9	83.8 ± 16.3	0.464
Internal rotation at 90° abduction	69.2 ± 6.2	72.6 ± 14.6	0.150
UCLA	28.8 ± 4.5	28.5 ± 4.5	0.780

Abbreviation: UCLA, University of California at Los Angeles.

**TABLE 2:** Basic information of patients before surgery

Basic information	Single‐row (*n* = 24)	Double‐row (*n* = 20)	*p* value
Gender (M/F, *n*)	12/12	15/5	0.09
Age at first dislocation (years)	24.8 ± 9.4	20.3 ± 5.2	0.100
Age at operation (years)	31.1 ± 10.6	26.7 ± 7.9	0.119
Dominant arm (*n*)	19	12	0.165
Glenoid bone loss (%)	10.7 ± 8.6	10.4 ± 7.6	0.934
Number of episodes of instability	9.6 ± 11.4	14.6 ± 21.4	0.186
VAS for pain	4.8 ± 3.2	5.0 ± 3.5	0.729
VAS for instability	7.7 ± 2.7	7.5 ± 2.4	0.606
ASES	61.8 ± 23.0	62.0 ± 23.5	0.951
UCLA	27.6 ± 5.5	29.5 ± 4.5	0.337
SSV	57.4 ± 24.9	59.2 ± 28.4	0.912

### Clinical Outcomes

For the single‐row group, the VAS for pain decreased from 4.8 in pre‐operation to 2.7 in post‐operation (*p* = 0.006) and VAS for instability also decreased from 7.7 to 1.9 (*p* < 0.001). A significant improvement in ASES was observed (from 61.8 to 83.2, *p* < 0.001). UCLA was enhanced from 27.6 to 33.8 (*p* < 0.001) and SSV increased from 57.4 to 86.3 (*p* = 0.001). The results of the double‐row group were as excellent as the single‐row. VAS for pain alleviated from 5.0 to 1.8 (*p* = 0.015). VAS for instability got a great relief (from 7.5 to 1.1, *p* < 0.001). The mean ASES score increased more than 20 (62.0 *vs*. 89.5, *p* = 0.001). UCLA rose from 29.5 to 33.5 (*p* = 0.006). SSV enhanced from 59.2 to 89.5 (*p* = 0.001) (Table 3). A comparison between the two groups can be seen in Table 3. There was no significant difference for VAS (for pain, *p* = 0.387; for instability, *p* = 0.345), ASES (*p* = 0.302), UCLA (*p* = 0.751), or SSV (*p* = 0.924), although the mean values seemed better in the double‐row group (Table 4). The recurrence rate of the single‐row group was 12.5% (3/24), while it was 10% for the double‐row group (*p*= 0.795).

**TABLE 3:** Comparison of clinical outcomes between before and after operation of each group

Clinical outcomes	Pre‐operation	Post‐operation	*p* value
Single‐row			
VAS for pain	4.8 ± 3.2	2.7 ± 3.0	0.006**
VAS for instability	7.7 ± 2.7	1.9 ± 2.2	<0.001***
ASES	61.8 ± 23.0	83.2 ± 17.4	<0.001***
UCLA	27.6 ± 5.5	33.8 ± 2.6	<0.001***
SSV	57.4 ± 24.9	86.3 ± 15.0	0.001***
Double‐row			
VAS for pain	5.0 ± 3.5	1.8 ± 2.4	0.015*
VAS for instability	7.5 ± 2.4	1.1 ± 1.0	<0.001***
ASES	62.0 ± 23.5	89.5 ± 12.5	0.001***
UCLA	29.5 ± 4.5	33.5 ± 2.9	0.006**
SSV	59.2 ± 28.4	89.5 ± 7.5	0.001***

Abbreviations: ASES, rating scale of the American shoulder and elbow surgeons; SSV, subjective shoulder value; UCLA, University of California at Los Angeles; VAS, visual analogue scale.

**TABLE 4:** Comparison of clinical outcomes between SR and DR group

Post‐operation	Single‐row	Double‐row	*p* value
VAS for pain	2.7 ± 3.0	1.8 ± 2.4	0.387
VAS for instability	1.9 ± 2.2	1.1 ± 1.0	0.345
ASES	83.2 ± 17.4	89.5 ± 12.5	0.302
UCLA	33.8 ± 2.6	33.5 ± 2.9	0.751
SSV	86.3 ± 15.0	89.5 ± 7.5	0.924

Abbreviations: ASES, rating scale of the American shoulder and elbow surgeons; SSV, subjective shoulder value; UCLA, University of California at Los Angeles; VAS, visual analogue scale.

### Range of Motion

Fourteen patients (31.8%) in the single‐row group and nine patients (26.5%) in the double‐row group were tested for active range of motion. The forward flexion, external rotation at the side, internal rotation at the side, and external rotation at 90° abduction did not have statistical differences. However, internal rotation at 90° abduction was significantly better in the double‐row group (48.9 vs. 76.7, *p* = 0.033) (Table 5).

**TABLE 5:** Comparison of range of motion between SR and DR group

Range of motion	Single‐row (*n* = 14)	Double‐row (*n* = 9)	*p* value
Forward flexion	168.6 ± 11.8	173.1 ± 12.1	0.369
External rotation at the side	46.1 ± 9.0	46.4 ± 6.4	0.734
Internal rotation at the side	62.5 ± 10.0	68.9 ± 2.2	0.159
External rotation at 90° abduction	87.3 ± 6.0	80.0 ± 19.8	0.829
Internal rotation at 90° abduction	48.9 ± 27.5	76.7 ± 24.0	0.033*

## Discussion

The main finding of this research was that the key point double‐row Bankart repair could achieve excellent clinical outcomes and a very low recurrence rate, although it didn't express significant advantages compared with single‐row repair in our cases. The key point double‐row repair still provided a good idea to combine the superiority of single‐row and double‐row suture. More stability and fewer anchor‐associated complications may support this new suture method.

### Key Point Double‐row Repair is a Useful Technique for Bankart Lesion

One of the advantages of our research was that it is a long‐term comparative study. Similar to our technique, Itoigawa *et al*.^9^ also reported a new suture method called 4 o'clock double anchor footprint fixation (DAFF). The difference was they used mattress sutures while bridge sutures were used in our study. Their fixation method was inspired by an anatomic research,^10^ which revealed the area of capsulolabral complex attachment on the glenoid neck wasn't even and there was a maximum adhesion region in the direction of 4 o'clock but this natural feature wasn't restored after repairing by a single‐row suture.^11^ They reported a short‐term result of 42 patients and the UCLA and Rowe scores got significant improvements. But they did not compare this with the single‐row method. From our long‐term comparison results, the key point double‐row suture could achieve no worse than single‐row suture, and the range of internal rotation at abduction seemed better.

### One Medial Anchor is Able to Provide Adequate Stability

The appropriate number of anchors in Bankart repair was still unclear. On the one hand, more anchors represent more stability. One review found the recurrence rate of Bankart repair decreased significantly when no less than four anchors were placed.^12^ Similarly, Bokshan *et al*.^13^ concluded from their cadaveric study that an additional 6 o'clock anchor could increase the fixation strength of Bankart repair with single‐row suture. The increased anchors could connect the capsulolabral complex and glenoid neck more tightly and provide more contact area for better union.^5^, ^6^ However, on the other hand, more anchors were also related to more complications and motion limitations. The number of anchors had been proven a high relation to arthritis in long‐term periods.^14^, ^15^ Many long‐term follow‐up studies reported 23% ‐ 39% of patients had mild to moderate arthritis after single‐row Bankart repair.^16^, ^17^, ^18^ Moreover, McDonald *et al*.^7^ discovered that Bankart repair brought an external limitation, especially for double‐row suture according to biomechanical tests. But there were no long‐term results of traditional double‐row repair. Our outcomes demonstrated only one additional medial anchor did not increase additional joint pain or limited range of motion.

Our results could also prove that only one medial anchor was needed to provide enough stability as the recurrence rate was as low as 10% and excessive medial anchors in traditional double‐row suture might contribute little to more stability. Many biomechanical studies about Bankart repair revealed that double‐row suture could recover more natural footprint area.^5^, ^6^, ^19^ But these found that the biomechanical parameters did not express superiority in the double‐row group.^19^ Particularly worth mentioning was Jonson who used just five anchors in double‐row suture as described by Lafosse *et al*.,^4^ and the footprint area coverage percentage was 73.4%, which did not shrink a lot compared with 86% reported by Ahmad *et al*.^5^ and 78% reported by Kim *et al*.,^6^ both of whom used eight anchors in the double‐row suture. Therefore, the excessive medial anchors did not contribute to recovering the footprint. This conformed to the cadaveric research by Itoigawa *et al*.^11^ as mentioned above.

At the last follow‐up, the internal rotation in the double‐row group was significantly better than the single‐row group. One hypothesis was that the medial anchor inserted to the anteroinferior glenoid helped to restore the stability of anteroinferior glenohumeral ligament at an early stage, which could provide extra support when doing internal rotation rehabilitation.

### Limitations

As a retrospective case–control study, there were some limitations. First, the rate of follow‐up loss was a little high (43.6%), partly because the follow‐up period was long and some patients' information had changed. Second, the short‐term follow‐up data was lacking, making it difficult to analyze the union rate after the operation. Third, we were short of radiological assessments after surgery and the last follow‐up. However, this was a long‐term controlled study, and all the surgeries were performed by one experienced surgeon to make sure the surgical techniques were consistent and the results were comparable. We look forward to more complete follow‐up results to supplement this study, especially the radiological data.

### Prospects of Clinical Application

The key point double‐row Bankart repair combined the utility of single‐row suture and the effectiveness of double‐row suture and avoided the potential complications. It could be one of the choices for anterior shoulder instability, especially for patients with critical glenoid bone loss who will have a high recurrence risk after receiving traditional Bankart repair. We hope there will be more clinical data to prove the effectiveness of our technique.

## Conclusion

As a new technique for Bankart lesion, key point double‐row suture uses only one additional medial anchor to achieve more stability compared with single‐row suture. And using one more anchor did not lead to limited range of motion. The long‐term outcomes also showed its utility. The low recurrence rate and potential biomechanical advantages making it a possibly superior choice to traditional Bankart repair.

## Ethics Statement

This study was performed at Department of Sports Medicine, Peking University Third Hospital. And this study was approved by the Peking University Third Hospital Medical Science Research Ethics Committee (No. M2022685). All methods were carried out in accordance with relevant guidelines and regulations. The informed consent was waived by the Peking University Third Hospital Medical Science Research Ethics Committee because the current study was retrospective.

## Author Contributions

XC and HW were two majors in data collection and writing the manuscript. YJ helped to polish the manuscript. ZS and GC designed this work.

## Funding Information

This study was supported by the National Natural Science Foundation of China (82172423 and 81871770), Beijing Natural Science Foundation (L222094 and 7222209). The funding source had no role in the design of the study and collection, analysis, and interpretation of data and in writing the manuscript.
